# Health needs, health care seeking behaviour, and utilization of health services among lesbians, gays and bisexuals in Addis Ababa, Ethiopia

**DOI:** 10.1186/s12939-019-0991-5

**Published:** 2019-06-11

**Authors:** Getnet Tadele, Woldekidan Kifle Amde

**Affiliations:** 1 Department of Sociology, Addis Ababa University, Addis Ababa, Ethiopia; 2 School of Public Health, University of the Western Cape, Cape Town, South Africa

**Keywords:** LGB, Health seeking behaviour, Heteronormativity, Intersectionality, Stigma, Ethiopia

## Abstract

**Background:**

Studies show that sexual and gender minorities have unique health care needs and encounter complicated problems to access health services. Drawing on the intersectionality approach, this paper examines the intersecting factors that determine health care seeking behaviour and utilization of health care services among Lesbians, Gays and Bisexuals (LGB) in Ethiopia including the diversity in experiences of these determinants and differences in the coping mechanisms to navigate these challenges within the LGB group. Despite the importance, there remains a paucity of evidence on the topic in Ethiopia.

**Methods:**

A concurrent mixed method design was used including survey of 100 LGB, and in-depth interviews and an FGD with 10 and 8 participants, each respectively. The quantitative data was analysed using descriptive statistics. Qualitative data was analysed thematically and triangulated with quantitative data.

**Results:**

The results show that heteronormativity intersects with LGB’s social position (sexual identity, social network and class) to influence health care need, health seeking behaviour or access to health services. Sexual health and mental health problems are main concerns of LGB, who reported to live under acute anxiety and fear of being exposed, or bringing shame and humiliation to themselves or their families. One of the main emerging themes from the research is the link between mental health and risky sexual practices. Risk perception to HIV was high among LGB, with two-thirds reporting high risk. Only 37.5% (33/88) stated being always motivated to seek care when sick and the rest cited the following barriers that stifled their health seeking behaviour and utilization of health care services: Stigma and discrimination (83%), shame and embarrassment (83%), fear of being discovered (78%), lack of LGB friendly services (45%), affordability (18%), distance (17%), and health care professional refusal (10%).

**Conclusion:**

Homophobia and criminalization of homosexuality, and heteronormativity of health care services intersect with LGB’s social position resulting in heterogeneity of risk, diversity of sexual and mental health needs, and difference in coping mechanisms (disadvantages and privilege). The main implication of the study is the need to recognize the existence of LGB and their diverse sexual and mental health needs, and link them to appropriate health care and pyscho-social services including HIV/AIDS prevention and treatment.

## Background

Moral discourses shape public attitude about what is acceptable and unacceptable, and spur actors to specific actions. Morality in sex and sexuality is a cultural and religious construct grounded in and often regulated by policy, legal instruments and ramifications [1]. The practice of same sex relations is both illegal and a social taboo in sub-Saharan Africa [2]. Sexual minorities have also received little attention, both in the realms of research and intervention.

Previous research has established that Lesbians, Gays, Bisexuals and Transgender (LGBT) community encounter multifaceted challenges that affect their health seeking behaviour and use of health care services. In the context of HIV/AIDS, addressing the unique health needs of at risk groups is reckoned as an effective strategy of tackling HIV/AIDS [3–5]. Studies conducted of late depict that LGBT in general and men who have sex with men (MSM) in particular experience high rates of HIV infection globally [4–6].

LGB in Ethiopia live under multifaceted social and legal strictures that mediate various aspects of their lives. The Penal Code, which underwent revision in 2005 after almost five decades, upholds criminalization of same sex relations. This in turn further reinforces the practice of religious institutions that vehemently oppose homosexuality. While local religious and other advocates against homosexuality are vocal and enjoy a great deal of support and public platforms, advocates for the rights of homosexuality are hardly visible except in cyber space, using pseudonyms, which appear to be the only safe space. These structural determinants inform attitude and behaviour of individuals, fostering stigma and discrimination and hate crimes against homosexuals both by citizens and members of the law enforcement (Tadele G: Under the cloak of secrecy: sexuality and HIV/AIDS among men who have sex with men (MSM) in Addis Ababa, unpublished) [7].

Same sex relation represents one of the least researched topics in Ethiopia wherein legal and social strictures against such relations pervade all aspects of life; and denial, stigmatization, and criminalization are the norm (Tadele G: Under the cloak of secrecy: sexuality and HIV/AIDS among men who have sex with men (MSM) in Addis Ababa, unpublished) [8]. The lack of data about men who have sex with men (MSM) and absence of interventions targeting this group have for long been glaring omissions in the national health reports [8–11]. Recent studies, however, underlined the existence of this group and the dangers of lack of recognition of the group in the HIV prevention and treatment endeavour (Tadele G: Under the cloak of secrecy: sexuality and HIV/AIDS among men who have sex with men (MSM) in Addis Ababa, unpublished) [11, 12]. The few studies on MSM in Ethiopia also emphasise the impact of structural factors that influence their access and use of health services. These studies also highlighted the dearth of data about politics of sexual identities of LGB, and state and extent of access to health services (Tadele G: Under the cloak of secrecy: sexuality and HIV/AIDS among men who have sex with men (MSM) in Addis Ababa, unpublished) [7, 8] in a context shrouded by ‘Homophobic public discourse and religious prohibition [and] … political silence’ (Tadele G: Under the cloak of secrecy: sexuality and HIV/AIDS among men who have sex with men (MSM) in Addis Ababa, unpublished).

This paper examines the intersecting factors that determine health care seeking behaviour and utilization of health care services among LGB in Ethiopia including the diversity in experiences of these determinants and differences in the coping mechanisms to navigate these challenges within the LGB group.

We draw on Intersectionality approach [13–16] to frame the research, and unpack differences in the lived experiences within and across categories of LGB. Intersectionality approach stresses how the existence of multiple factors mutually construct one another and further illuminates how individuals are positioned in unequal power relations [13–16]. According to Hankivsky et al. (2014) ‘social locations are inseparable and shaped by interacting and mutually constituting social processes and structures, which, in turn, are shaped by power and influenced by both time and place’ [13], p., 2. We argue that interrogating the lived experiences of LGB is pivotal to understand factors influencing health-seeking behaviour and utilization of health services.

## Methods

### Research design and sampling

A concurrent mixed method design (Meissner et al. 2011; Creswell 2009) was used with the purpose to advance understanding about health care seeking behaviour and utilization of health care services among LGB in Ethiopia. Secrecy and criminalization, coupled with widespread stigma make identifying and recruiting LGB participants in Ethiopia an onerous task (Tadele G: Under the cloak of secrecy: sexuality and HIV/AIDS among men who have sex with men (MSM) in Addis Ababa, unpublished) . As a result, purposive sampling was found appropriate to identify participants [17, 18]. The first author came to know another Ethiopian researcher who recently conducted a study on MSM and this person put the researchers in touch with contacts, both personal and virtual as entry to identify and recruit potential participants who are in same-sex relations. This was followed by the use of snowball sampling wherein initial contacts were asked to recommend other potential participants for the study. Though it was mixed method research, quantitative findings are by no means representative, due to the procedure used to recruit participants and size of the population.

### Data collection

The development of the research tools (survey questionnaire and interview guides) was informed by review of literature and the purpose of the research. The questionnaire was pilot tested and revised. In the quantitative wing of the research, 100 LGB took part in a survey using interviewer or self-administered structured questionnaire. Among others, the main issues covered by the questionnaire include: lived experiences of LGB with specific focus on social networks, health needs, health care seeking behaviour, barriers, and coping mechanisms. Five gay Ethiopian men were recruited as research assistants (Four data collectors and one supervisor). They all had first degree and above in social sciences, and the first author provided them with short training on research methods and data collection in general and the data collection instrument in particular.

In the qualitative arm of the research, individual and group interviews were used to generate data about the lived experiences of LGB in relation to their social networks, health needs, barriers to health care services, and ways in which they navigate these challenges. The in-depth interviews with 10 MSM and an FGD with eight MSM were carried out by the first author of the article. It was not possible to find willing Lesbian study participants for the in-depth interview or FGD. This is perhaps due to the fear of exposure and grave implications in their lives. The in-depth interviews and focus group discussions were audio-recorded with the permission of participants. The data collection was undertaken in November and December 2017.

### Data analysis

The quantitative data was captured and analysed using SPSS. Seven of the 100 questionnaires had major gaps and thus they were removed. The qualitative data was analysed thematically with themes generated from research questions and data. The qualitative audio recorded in-depth interviews and focus group discussion were transcribed and translated into English by the research assistants, which were then cross checked by the authors for accuracy and completeness. ATLAS, a qualitative data analysis software, was used for data analysis. The transcripts were open coded by the first author. Through an iterative process, categories were developed from the codes. The categories were further developed into broader and more analytical categories/themes that respond to the research objective [19]. The data was triangulated combining the information from different methods and sources [18].

## Results

### Profile of participants

Those informants who participated in in-depth interviews and FGD were between the ages of 23 and 32 years, and they represented a diversity of ‘sexual identity’, ethnic groups, beliefs and socio-economic background. Some of them were graduates from the university and involved in a variety of professional activities. There were also school drop-outs and school leavers without any gainful employment.

The quantitative survey covered ninety-three LGB with a median age of 26 (Minimum 18, Maximum 42, Standard Deviation 4.75). The vast proportions of the participants were originally from Addis Ababa and reside in the city (80.6, and 97.8%, respectively). With respect to their sexual identity 64.5% (60/93) identified themselves as gay, 18.3% (17/93) bisexuals, and the remaining 17.2% (16/93) were lesbians.

Close to half of the participants (48.4%, 45/93) were Orthodox Christians, followed by Protestants (12.9%, 12/93), Muslims (12.9%, 11/93) and Catholics (3.2%, 3/93). A significant proportion of the participants (20.4%, 19/93) did not specify their religious membership. For predominantly religious society, this perhaps has to do with the dissonance that the LGB feel between the normative prescriptions of their religion and their sexuality. In-depth interview informants alluded to experiencing cognitive or emotional dissonance towards religion because of their ‘unacceptable’ sexuality.

‘Right now, I don’t practice any religion. I am agnostic. But I grew up in a protestant Christian family.’ 30 years old male interviewee.

‘ … it [your sexuality] will distance you from your religion and spiritual life. … after I got into this life, I stopped going to church. This life makes you a nocturnal person.’ 25 years old male interviewee.

With respect to education, half of the participants had first degree or above (54.8%, 51/93), about one in four (24.7%, 23/93) had Technical and Vocational Education and Training (TVET, i.e. 10+ three or diploma), and the rest completed high school (17.2%, 16/93).

Employment wise, close to half (43.0%, 40/93) were full time employed. The rest were studying (15.1%, 14/93), self-employed (19.3%, 18/93), part time employed (9.7%, 9/93), or unemployed (8.6%, 8/93).

The vast majority of the participants were single (86.8%, 79/91), with the remaining being married (7.5%, 7/93), cohabiting (3.2%, 3/93) or widowed (2.1%, 2/93).

### Coming out and reactions

The survey data shows variations in the extent to which LGB disclose their sexuality to individuals in their various social groups (Table 1).

**Table 1:** Disclosure of sexual orientation

Disclosure	Gays (*N* = 60)	Lesbians (*N* = 16)	Bisexuals (*N* = 17)	Total *N* = 93
Family	25%	18.7%	11.8%	21.5%
Friends	45%	31.2%	41.2%	41.9%
Work colleagues	1.6%	0.0%	17.6%	4.3%
Online	78.3%	37.5%	64.7%	68.8%

As depicted in the above table, friends were the most reported (41.9%, 39/93) followed by family members (21.5%, 20/93), or work colleagues (4.3%, 4/93). Seven in ten LGB (68.8%, 64/93) claimed to be open about their sexual orientation on online spaces like social media, where they still maintain anonymity by using pseudonyms.

Disclosure, wilful or not, was often met with stigma, discrimination, rejection, isolation, verbal and physical abuse. The responses obtained from the interviews and FGD describe the serious repercussions of coming out, wherein LGB experience negative reactions including being disowned by their families, facing violence, or shame.

“In Ethiopia, everyone perceives this thing [same sex sexual relation] as something outrageous. … People’s perception forces us to hide and live in frustration. … But since we can do nothing about it, we are just living it, if we call this living”, FGD informant 2.

“… the idea of mingling with other people feels like death to us. It’s only amongst ourselves that we socialize. Whenever we socialize with other people, there is stigma, discrimination and stern faces. The fear of this has forced us to be segregated in great numbers in one place; hidden. … The attempt to socialize with the society by itself feels like a great struggle.” FGD informant 5.

As one FGD participant noted, the media reinforces stigma and discrimination against LGB community.

“All written documents [on media outlets] are negative about the ‘Zega’^1^ [LGB] community. Nothing positive is being written and community has negative attitude about the ‘Zega’ community.” FGD informant 1.

The quantitative and qualitative data show that many LGB were adamant that they would not consider coming out in the current social context, and they live under acute anxiety and fear of being exposed or bringing shame and humiliation to themselves or their families.

“I haven’t told anyone and I am not willing to do so. … if you tell one person that person would tell another and before you know it everyone will know about it. … I worry about my family. I don’t want people to point fingers at my parents and say his or her son is that way or this way.” 30 years old interviewee.

“If my family knows, … I think they would throw me out of the house … I would lose them forever; and my family means everything to me.” 23 years old interviewee

### Risky sexual practices and risk perception

The LGB in the survey rated their risk of HIV infection to be predominantly high. Six in ten respondents (55.9%, 52/93) rated their level of risk to be 5 and above (with 10 being high and 1 low). The vast majority of the respondents (86.0%, 80/93) also perceive LGB community to be more vulnerable to HIV/AIDS infection than the general community.^2^ Close to three-quarters of the LGB (72.0%, 67/93) reported to having had unprotected anal sex. The reluctance to use protection and the underlying reasons are further highlighted in the qualitative responses, as narrated by the two research participants below.

‘I have had unprotected sexual relationship with two of my friends who died of AIDS. It’s like a loop and many people have somehow been sexually connected.’ 23 years old interviewee.

‘Because I have had lots of unprotected sex previously, I don’t see the use to start [using condom] now and I might already be [HIV] positive.’ 32 years old interviewee.

Fourteen percent (13/93) of the LGB reported to be in committed monogamous relationship. The results for the various groups were 15.0% (9/60) gays, 6.2% (1/16) lesbians, and 17.6% (3/17) bisexuals. Considering the criminalising context, fraught with stigma and discrimination, it is significant that 14% of LGB were in a committed same sex monogamous relation.

According to participants of the qualitative research, while the practice of having multiple sex partners was common among LGB, maintaining a committed relationship was perceived to be very hard. The responses imply that some LGB have accepted the inevitability of contracting HIV/AIDS due to their exposure to multiple partners and risky sexual practices. A 25 years old interviewee noted:

“I do not think I will be shocked if I am told that I’m HIV positive. I know what I had done and my practices. Whenever I go for testing I go with the notion that the virus is in me,”

Some participants implied that what contributed to unsafe sexual practice among LGB is the lack of safe social space for same sex sexual relation, and the pressure to make the most of chance encounters.

‘I have multiple sex partners. I don’t know why but when people meet and are attracted to each other, there is no problem or worry to have sex. The act doesn’t bother us that much. Actually, what worries us much more than having sex is where to do it.’ 25 years old interviewee.

### Accessing and using condoms and lubricants

About three-in-ten respondents (31.2%, 29/93) reported to use condom consistently. The rest indicated using occasionally (54.8%, 51/93) and not at all (11.8%, 11/93). The qualitative data illuminate the reasons for inconsistent condom use, which include fear and shame associated with buying condoms, sense of urgency and lack of preparedness, being under the influence of alcohol, preference to have sex without condom with the intent to improve satisfaction, or selective non-use of condom with supposedly ‘safe or trusted’ partners. The following quotes reflect the range of reported reasons for unprotected sex:

‘Sometimes my mates are not willing to use condom; they just don’t like it.’ 26 years old survey participant.

‘When I use condom I don’t get the same satisfaction.’ 27 years old survey participant.

‘It’s very difficult to find condoms on a regular basis.’ 27 years old survey participant.

‘I only wear condom when I don’t trust the other person.’ 23 years old survey participant.

‘I am scared to buy condoms from shops.’ 25 years old survey participant.

About two-thirds of the LGB (65.6%, 61/93) reported to use lubricants. The figures vary across the groups: 68.3% (41/60) gays, 50.0% (8/16) lesbians, and 70.5% (12/17) bisexuals.

The qualitative interviews reveal further information in relation to what type of lubricant participants use. Many claim that due to the difficulty of accessing water based lubricants, they resort to using easily accessible oil based lubricants, which may damage the condom. According to some respondents, even in instances when water based lubricants were available in selected pharmacies, marketed for the general population, LGB still shun buying them for fear of giving away their sexual identity, and possible negative reactions. A few LGB stated getting the lubricants from LGB peers that travel abroad.

‘Even when they were selling lubricants in pharmacies, many [LGB] preferred to ask people to bring us some from abroad. They were not comfortable to buy from pharmacies because the looks and reactions you get from the pharmacists was scary.’ 30 years old interviewee.

The above quote shows the negative reactions of pharmacists deters LGB from accessing lubricants. Thus, accessing lubricants was found to be safer and possible mainly through networks of peers who travel abroad, or who informally work with certain embassies in the city around. The response below directly relates to this point:

‘I personally get lubricants from friends. I have friends who get lubricants from embassies. They work on health related matters regarding men.’ 25 years old interviewee.

The FGD participants similarly noted the difficulty of buying lubricants from pharmacy, and suggested the relative ease of accessing these products in events organized secretly by LGB.

### STIs/HIV testing

Three in ten respondents (30.1%, 28/93) reported contracting Sexually Transmitted Infections (STIs) in the past. The figures vary across the different groups: 33.9% of gays, 23.5% of bisexuals, and 25% of lesbians. Testing for HIV was reported to be quite common, cited by 82.8% (77/93) of the respondents (79.7% of gays, 100% of bisexuals, 81.3% of lesbians) though responses to such questions are likely to be socially desirable. Fear of positive diagnosis was cited by LGB that reported not doing the tests.

The qualitative study found two contrasting findings with respect to testing practice for STI and HIV. Some LGB reported that with growing recognition of their risky sexual practices, they are anxious and get tested regularly, while others reported being too anxious to get tested.

‘I have been infected with STDs two three times previously. I was very scared, especially the first time. I thought I was also infected with HIV. … I do get tested for HIV every three months.’ 25 years old interviewee.

‘I have never been tested because what if I am HIV positive? What will I do if the test comes positive? That scares me. … I fear it will devastate me. Whenever something [illness] happens I am living in fear [thinking] that it is because of the virus …. I lose my self-worth and confidence.’ 32 years old interviewee.

In the focus group discussion, participants noted the importance of peer support in dealing with sexual health problems, wherein they exchanged advice about STI treatment or coping with mental health issues, information about access to treatment, condoms or lubricants.

‘If I experience some anal infection, I will ask my friends for any advice. Some of them often recommend [using] hot water mixed with salt etc. … Mostly we use traditional medicines.’ FGD informant 2.

‘We only ask tips from each other: ‘what do you do for this and that?’, ‘what do you use to get rid of this and that?’ …No one wants to go to a health facility’ FGD informant 1.

### Mental health, substance abuse and risk behaviours

LGB’s mental health need in the context of risky sexual practices has emerged as one of the more significant themes in this study, particularly in the qualitative data, wherein mental health was perceived as a major health needs among LGB.

Quantitative data show that some LGB experienced depression (10.7%, 10/93) and stress (14%, 13/93). The other reported mental health problems include anxiety and panic attacks (5.3%, 5/93), self-harm (3.2%, 3/93), eating disorder (3.2%, 3/93), and suicidal ideation (2.1%, 2/93).

The qualitative data highlight lack of care to address the mental health problems, and negative implications. Data also illuminate that the mental health problems LGB experience are related to external stigma and discrimination, real or anticipated, and internalized stigma, as noted by the following participants.

‘Even if I always think and enjoy my sexuality, sometimes I feel like I’m wrong and abnormal and it stresses me.’ 20 years old survey respondent.

‘Sometimes I have suicidal thoughts due to chronic stress.’ 26 years old survey respondent.

‘Even when someone stares at us we automatically suspect that he/she might know us. … The stress is so heavy to the extent that some of our friends have lost their mind forever and ended up on the street.’ FGD informant 7.

‘The major reason why HIV is more rampant in our [LGB] society than others, in my opinion, is carelessness. And this carelessness comes from the value the society gives to us. … we face stigma and discrimination over and over again and experience stress, frustration and mental illness.’ FGD informant 5.

The above qualitative responses suggest the link between risky sexual practice and mental health. Depression was reportedly caused by social stigma and not being allowed to live openly.

LGB reported to deal with mental health issues such as depression, by indulging in alcohol, Khat, or cigarette use. The survey data show wide use of alcohol, Khat and Cigarettes among LGB (Table 2).

**Table 2:** Cigarette, Khat, alcohol, and non-prescription drug consumption among LGB

Consumption	Gays (N = 60)	Lesbians (N = 16)	Bisexuals (N = 17)	Total N = 93
Smoke cigarettes	48,3% (29/60)	50,0% (8/16)	64,7% (11/17)	51,6% (48/93)
Chew Khat	60,0% (36/60)	43,8% (7/16)	64,7% (11/17)	58,1% (54/93)
Drink alcohol	86,7% (52/60)	68,8% (11/16)	82,4% (14/17)	82,8% (77/93)
Non-prescription drugs	11,7% (7/60)	0,0% (0/16)	11,8% (2/17)	9,7% (9/93)

As the above table shows, alcohol was the most used by the group (82.8%, 77/93), followed by Khat (58.1%, 54/93) and cigarettes (51.6%, 48/93). A small group of LGB also reported using non-prescription drugs (9.7%, 9/93). There were differences in drug use practice among participants, with chewing Khat and drinking alcohol less common among women than men. The survey data does not indicate whether participants misuse or excessively use these items, which are all legal in Ethiopia.

The qualitative data, however, suggest the link between excessive use of alcohol and unprotected sex among LGB. Statements such as ‘We were both drunk and had sex without condom’ or ‘Most of the time I have sex after I get drunk, and I forget protection’ were not exceptional among informants.

### Health care seeking behaviour, health service utilization and barriers

Only 35.5% (33/93) of the respondents reported that they are always motivated to seek care when they are not feeling well. Differences exist among the different groups, bisexuals (58.8%), followed by gays (30%, 18/60) and lesbians (31.2%, 10/16).

A significant proportion of the respondents (58.1%, 54/93) stated that they are only motivated sometimes, and reported not being consistent in their health care seeking behaviour due to fear of homophobic reaction (18.5%, 31/54), confidentiality concerns (42.6%, 23/54), or fear of being stigmatized (35.2%, 19/54) (Table 3).

**Table 3:** Barriers to accessing health care

Barriers	Gays (*N* = 60)	Lesbians (*N* = 16)	Bisexuals (*N* = 17)	Total *N* = 93
Getting money needed	21,7%	12,5%	11,8%	18,3%
Fear of being discovered	80,0%	68,8%	82,4%	78,5%
Fear of stigma and discrimination	86,7%	68,8%	82,4%	82,8%
Being ashamed/embarrassed to seek service	85,0%	81,3%	76,5%	82,8%
Lack of convenience at the health facilities	45,0%	62,5%	29,4%	45,2%
Health care professional refusal	10,0%	6,3%	5,9%	8,6%

The table above depicts that fear of stigma and discrimination was the most reported barrier, cited by 82.8% (77/93) of the survey participants. Being ashamed or embarrassed to seek service was equally the most reported constraint, mentioned by 82.8% (77/93) of the LGB survey participants. This was corroborated in the qualitative data.

‘I didn’t go to the doctor because what am I going to say? There is no one who would treat you if you said this [anal infection] happened when you were having sex. So you stay home tolerating the pain.’ 23 years old interviewee.

For the majority of the LGB (78.5%, 73/93) in the survey fear of being discovered/outed was a hurdle to access health care when they are sick or when they seek medical advice or treatment. The responses in the qualitative data shed light about the ambivalence LGB face towards being present in public spaces, or being open about their sexuality to health professionals.

‘[The] fear of stigma and homophobic reactions … is paralyzing. … Our society doesn’t hold back from throwing homophobic slurs purely based on the way you walk, talk or dress. Imagine how terrifying it would be to face a health professional when you have an [anal] infection that would automatically expose your sexual orientation.’ 30 years old interviewee.

Lack of convenience at the health facilities was mentioned by close to half the LGB survey participants (45.2%, 42/93) as a constraint to access health care when they are sick or seek medical advice or treatment. Responses from the in-depth interviewees also reiterated the same:

‘ … because we don’t have sensitized doctors in our country, you can’t go to hospital. Even if you go, what would you say? Because you can’t tell them [your sexual practice because if you do] they won’t treat you. So we don’t go. Whether we are sick or whatever, we stay home.’ 23 years old interviewee.

In the event of sexual health problem, LGB stated that they seek support mainly from friends (58.1%, 54/93), followed by public health facilities (31.2%, 29/93), private health facilities (47.3%, 44/93), public pharmacies (9.6%, 9/93), private pharmacies (5.4%, 5/93), or holy water/traditional healer (2.1%, 2/93). Only one in ten of the LGB (9.7%, 9/93) consider the services to be convenient or LGB friendly, while it is the opposite for the rest. Those with the positive review claimed that they are content as long as they are able to get the services.

The qualitative interviews shed further insight about LGB’s preference as to where they access health services, which include convenience and ease of concealing sexual identity. Their notion of convenience seems to be linked with the risk of being outed or being obliged to disclose their sexual practice.

‘I think public health facilities are better. They see and treat a lot of people every day and they do not pay that much attention to their patients. That will give you the chance to be unnoticed. But private health facilities pay too much attention and they are more concerned about their image and the centre’s reputation.’ 25 years old interviewee.

‘Public health care providers don’t have time to ask for details. So that’s very convenient.’ 28 years old survey respondent.

Getting money needed for treatment was another challenge to access health services according to about one-fifth of the participants (18.3%, 17/93). Financial challenges among the LGB were also linked with unemployment and mental health problems. In the qualitative interviews, participants mentioned sense of comradeship among LGB during times of crisis.

‘We have had to raise money so that some people could get medical treatment. There are many people [LGB] who are addicted [khat, alcohol, cigarette] and don’t work. Others are incapable of working because of mental health issues.’ 30 years old interviewee.

Distance to health facility was reported as a barrier to access health by relatively smaller proportion of the survey participants 17.2% (16/93). In the qualitative data some LGB reported choosing to seek health care outside their community to avoid being recognized or exposed. This has transport cost implications, which may vary depending on distance to target health facility and whether they access service in private or public health facilities. The above analysis shows that health seeking behaviour intersects with the LGB’s geographic location, financial capacity and a number of other attributes.

The qualitative data further suggest that social networking, which seems to emerge from or enabled by financial or educational capacity, provides leverage to some LGB to access treatment, in the form of having information or being referred by LGB peers to a friendly health care professionals.

Of all the barriers mentioned by LGB, the negative attitude of health care professionals was emphasized as a significant hindrance to access health care services. Some of the responses from the interviews and FGD shed light on how LGB experience health facilities as discriminatory spaces.

‘I know people who had STDs from behind [anal] and when they go to health facilities they were surrounded by all the nurses and doctors and they were humiliated.’ 30 years old interviewee.

A closer look on relation the LGB participants had with health care professionals show that only 14.0% (13/93) of the LGB claim to be open about their sexuality with health care providers (nurse/doctor). The remaining vast majority 86% (80/93) emphasized that they conceal their sexuality. The later group cited multiple reasons including fear of homophobic reaction (81.25%, 65/80), fear of being stigmatized (40%, 32/80), confidentiality concerns (31.2%, 25/80), and past negative experiences (2.5%, 2/80).

The qualitative data also show that LGB dread getting questions about their sexuality from health care providers, and reported that when confronted with such questions they often deny or leave the facility altogether without getting service. The following excerpts illuminate these encounters.

‘I once told the doctor that I am gay then he started advising me to stop [same sex relation] then I said [to him] I can’t. Finally, he told me it is useless to treat me.’ 20 years old survey respondent.

‘Once I went to see a doctor and as I was explaining my symptoms, he suspected that I was gay and he told me that they don’t provide service.’ 24 years old survey respondent.

‘There is a … doctor who knows that I am gay and very friendly and when I went to the health centre where he works, the nurse told me he was not there when he was there.’ 23 years old survey respondent.

The qualitative results show that due to the heteronormative context the aforementioned access barriers seem to be more pronounced among LGB that have receptive anal intercourse, and experience anal fissures or STI of the anus and rectum. The barriers were reported to be less pronounced for men that have insertive anal intercourse and experience STI around their penis. The barriers are manageable for the later group that practice insertive intercourse, as they can explain away their situation by suggesting that they get the infection through heterosexual relation, from female sex workers or girlfriends:

‘I went to a health centre and saw a doctor. … He then told me it is STI and I need to bring my girlfriend. Then I told him … she is a sex worker and I don’t know her. … The doctors don’t have any means to know from whom you got the infection. You just tell them it’s with a woman.’ 25 years old interviewee.

In contrast, for those who had anal infections due to receptive intercourse, seeking treatment from health professionals is a daunting challenge. They evade questions about sexual practice or make up false reasons, or even leave health facility without treatment in the event that they feel health care providers are suspicious. The excerpts below demonstrate such experiences of participants.

‘Anyone could get STDs. But this one [anal infection] is internal that is supposedly caused by this [receptive anal intercourse] alone. … To avoid getting help from them [medical professionals], many die hiding in their homes.’ FGD informant 4.

‘I once went to private hospital … the doctor did some tests. Then he said that I had intestinal infection, and he asked me whether I lifted heavy objects or did strenuous activities, I said that I didn’t …. Then he continued asking me ‘ … .or are you … .’ At that time, I got up and left the room without any medicine fearing the words he was about to say.’ FGD respondent 6.

‘ … We usually face various infections on the intestine … Since there is no adequate medical care, and since there is no way to go openly into a medical institute to get help, the conditions will gradually complicate and lead to death. So people just sit around and wait …. ’ FGD respondent 8.

One 25 years old male interviewee reported being raped, ‘I told him [the doctor] that I went to a party and I got drunk and this happened [rape]. Another informant presented the following made up story:

‘I had a tearing while having sex and had to see a doctor. When the doctor asked me what happened, I told him that I had an accident – “a chair broke and the metal leg hit me on that spot [anus].” … He believed me and didn’t ask me any further questions. I got treated and left.’ 23 years old interviewee.

Another significant theme emerging from the qualitative data is that while concealing the causes of one’s illness from health care professionals helps LGB avoid stigma, shame and discrimination; it, however, led to misdiagnosis of their condition by health professionals, and possible complication.

‘I could not tell them the real reason so they told me I have another disease. They said it was typhoid or typhus but I knew what happened to me. But I took the medicine they prescribed for me. I took it for a while and stopped. After that I stopped having sex for a long time. I was very sick,’ 23 years old interviewee.

The qualitative data also highlight experiences of LGB wherein the health professionals despite being aware of same sex sexual practice leading to the health problem, they go about caring for the LGB without any judgment.

‘I know of a friend who had a lesion around his anus. There was bleeding. He said he went to the doctor and … said it was haemorrhoids. The doctor could tell it was not [haemorrhoids] and told him … it was caused by something forceful. He asked him if he was raped. He replied he was not raped then the doctor said if you were not forcefully raped that means you did it willingly. The doctor did not make a big deal out of it and treated him after all.’ 25 years old interviewee.

These were the kinds of health professionals that few LGB previously consider friendly and recommend to peers in their social network to visit for treatment.

## Discussion

We believe that the study contributes to understanding of the diversity in the experiences, needs and risk behaviour of LGB in Ethiopia. Drawing insights from intersectional approach, the research attempts to shed light on two recurring themes in the data: heterogeneity of LGB health needs and risk behaviours, and heteronormativity of health services.

### Heterogeneity of LGB health needs and risk behaviours

LGB do not have a homogeneous experience when it comes to discrimination, stigma, and prejudice. This was evident in our analysis that many LGB research participants reported varying experiences; particularly with respect to sexual and mental health needs and access and uptake of health services. The intersecting factors that gave rise to heterogeneity of experiences include nature of health complication, financial capacity, educational status, and social network.

Health complications related to anal infections generate a great sense of stigma, internal and external. LGB experiencing such infection find it hard to seek or access proper treatment, and often end up being misdiagnosed or using traditional/alternative treatment. This is alarming considering the high prevalence of unprotected anal sex reported among LGB participants (72.0%, 67/93). Studies on MSM in Addis Ababa noted the great sense of stigma surrounding anal sex and the absence of any health information or services. The studies contend that interventions that recognize diversity of sexual practices and identities are long overdue and imperative to avert the multifaceted implications of denial and neglect of such at-risk and marginalised group [7, 11].

Our study demonstrates that financial privilege and social capital give LGB leverage to navigate access barriers to condoms, lubricants, and health treatment. LGB who travel abroad, or have links with international agencies had better access to lubricants and condoms, which they share with peers in their social network.

The study shows that LGB experience multiple mental health problems living under strict social scrutiny and fear of being outed or bringing shame and humiliation to themselves or their families. Sense of shame or stigma in the event of being exposed is so strong that a few LGB mentioned ideation of suicide and fleeing. This has resonance with previous studies [3, 21, 22] that established how stigma and discrimination, perceived or real, that is closely associated with being LGB take their toll on the mental health of members of this group. Mayer noted ‘Mental health disorders are not inherent to being a sexual minority person but can manifest as a result of leading marginalized lives, enduring the stress of hiding one’s sexuality, or facing verbal, emotional, or physical abuse from intolerant family members and communities.’ [3], p., 991. Describing the way internalized stigma operates, Meyer stated ‘ … even if one’s minority status is successfully concealed, lesbians and gay men may be harmed by directing negative social values toward the self.’ [21], p., 14.

Stahlman et al. also argued ‘Social stigma is common among men who have sex with men (MSM) across Sub-Saharan Africa, and may influence risks for HIV and sexually transmitted infections (STIs) via its association with depression’ [22], p., 1460. A study in Ethiopia entitled ‘Heteronormativity and “troubled” masculinities among men who have sex with men in Addis Ababa,’ found widespread and deep dissatisfactions among the group about their sexual identity spurred by the sense of transgressing long standing societal norms [23].

Our analysis shows prevalence of mental health conditions among LGB closely related with their sexuality, and for which they have not received any professional care or advice. The results also show that LGB’s experience of mental health problems depends on their social network and support they receive. LGB rely on peer social groups (virtual or physical) for social, financial and emotional support.

Despite the class difference, none of the research participants mentioned seeking mental health services. This mirrors the state of mental health issues and mental health care services in the county, which is insignificant considering the staggering magnitude of the problem. The country’s mental health strategy corroborates that ‘mental illness is the leading non-communicable disorder in terms of burden. … [Evidence suggests that] mental illnesses have been overlooked as a major health priority in Ethiopia and other Low and Middle Income Countries (LMICs), and underscore the need for public health programs targeting mental illnesses’ [24], p., 9. It is safe to argue that the widespread misperception and stigma associated with mental health in the country intersects with sexuality to further marginalise LGB from accessing mental health service.

### Heteronormativity of health care services

Ethiopia is one of the countries in Africa that criminalise homosexuality. This entrenches the notion that sexual identity and performances are within the parameters of heterosexual identity. The moral discourse around family and relationships are also encapsulated within heterosexual values and beliefs (Tadele G: Under the cloak of secrecy: sexuality and HIV/AIDS among men who have sex with men (MSM) in Addis Ababa, unpublished) [7, 11, 23]. This study clearly shows that heteronormative beliefs and values are ingrained in the health system of Ethiopia at various levels. This aligns well with a growing recognition of health systems in the literature as social constructs themselves wherein societal norms mediate attitude and practices of policy makers, patients, and health care providers, and health outcomes (Tadele G: Under the cloak of secrecy: sexuality and HIV/AIDS among men who have sex with men (MSM) in Addis Ababa, unpublished) [25, 26]. This was apparent in this study that heteronormativity greatly influences perception and behaviour of both LGB and health care providers. Participants are reluctant to disclose their sexuality when accessing health services because of the widely prevalent heteronormative social values. LGB participants expressed their mistrust towards health professionals, whom they found or expect to be judgmental, discriminatory, and insensitive. Literature depict that trust is an issue even in contexts where same sex relation is legal and services are available [27] and LGB ‘continue to interact with a health care system that is unaware, insensitive, and unprepared to meet their needs’ [3], p., 992.

Globally, a review of literature highlights wide prevalence of heteronormativity among health care professionals, wherein they tend to assume their clients to be heterosexuals, or if they suspect about their homosexuality, then they tend to engage with them in terms of their sexuality, disregarding their many other needs [15]. A qualitative study on GPs in UK found that homophobic attitudes and lack of awareness about sexual practice of LGB inhibited health professionals from discussing sexual health issues [28]. The situation is bound to be far worse in Ethiopian context, and represents missed opportunity for testing, and linking to care.

It was evident in the study that embedded in a context that considers same sex sexual practice as a deviant behaviour in the society, and heterosexuality as a normal behaviour, LGB with sexual health problems compromise and hide their identity to access medical attention from the health care professionals. Previous studies found that stigma and discrimination, actual or imagined, permeate the lives of sexual minorities and negatively influence their use of health services [3, 8, 29, 30].

As argued by our informants, the lack of transparent communication between LGB patients and health care professionals has led to misdiagnosis of illness, and lack of treatment. This is a significant result demonstrating the multiple layers and facets of the challenges facing LGB. Report by Mayer et al. (2008) found similar negative patterns in relationship between MSM and health care professionals. ‘LGBT patients have multiple reasons for not disclosing their sexual or gender identity to providers … [to] the extent that these concerns cause LGBT patients to delay receipt of care or withhold information that may be important to treatment, effective medical care can be compromised.’ (3992).

### Limitations

We acknowledge that the findings from this study, while they help advance understanding of the experiences of LGB, they are not representative of the experiences of LGB across the country. This has to do with the way participants were selected, the geographical focus on Addis Ababa, and the limits that we were only able to include LGB that were privy to the networks we managed to access. Furthermore, we are using LGB as an identity of sexual minorities and acknowledge that the research has not deeply engaged with Lesbians and it did not include transgender and intersex people because of difficulty of accessing them. The study is confined to exploring contextual realities of LGB community in Ethiopia mainly with respect to health seeking behaviour, and has not addressed the totality of LGB experience and the nuanced impact stigma and criminalization have on other spheres of their lives.

The authors are two Ethiopian heterosexual men with public health and social science background. The first author has done extensive work on MSM over the years. Being raised and lived in a conservative and heteronormative country such as Ethiopia, where LGB are hardly visible, we acknowledge the limits of our understanding of the experiences of LGB. We recognise that our positionality as researchers might generate a power dynamic that influence the richness of the research and ability to unpack multi-layered socio-political issues. The strength of this article is that we used insights from intersectionality approach to explore and unpack disadvantages and privileges, and illuminate differences in the lived experiences within and across categories of study participants [13–16] and the factors that affect access and utilization of health care.

## Conclusion

Social and legal strictures against homosexuality coupled with widespread heteronormativity in the health system make LGB one of the most marginalized at risk groups in Ethiopia. Differences in LGB’s social position (nature of sexual health complication, finance, education, social network) result in heterogeneity of risk, diversity of sexual and mental health needs, or difference in coping mechanisms (disadvantages and privilege).

The multiple mutually reinforcing barriers LGB face to access health services demonstrate the need for concerted action, and importance of addressing the underlying challenges posed by heteronormativity in the health system and among health care professionals and LGB. The main implication of the study is the need to recognize the existence of LGB and their diverse sexual and mental health needs, and link them to appropriate health care and pyscho-social services including HIV/AIDS prevention and treatment. We hope that the paper lays the ground for other studies to further explore LGB communities’ lived experiences.

## Data Availability

Not applicable.

## Abbreviations

AIDS: Acquired Immune Deficiency Syndrome
CMI: CHR Michelsen Institute
FGD: Focus Group Discussion
GPs: General practitioners
HIV: Human Immune Virus
LGB: Lesbians, Gays and Bisexuals
LMICs: Low and Middle Income countries
MSM: Men who have sex with Men
NGOs: Non Governmental Organizations
SPSS: Statistical Package for Social Sciences
STIs: Sexually Transmitted Infections
TVET: Technical and Vocational Education and Training
UK: United Kingdom
UNAIDS: The Joint United Nations Program on HIV/AIDS

## Footnotes

1 The Amharic term ‘Zega’ translates to ‘citizen’. It is commonly used among the LGB population to covertly refer to themselves, and has a positive connotation in contrast to several other local terms that are pejorative. The term further got popularity through being used as a handle/hashtag among Ethiopian LGB in the social media.

2 According to the HIV Prevention in Ethiopia National Road Map 2018–2020 report, “Seven out of the nine regional states and two city administrations have HIV prevalence above 1%. Prevalence in Addis Ababa is 3.4%. In 2017, there were an estimated 613,000 people living with HIV, of whom 62% female.” [20], p., 4
